# Supplementary material for: ADAM17 selectively activates the IL‐6 trans‐signaling/ERK MAPK axis in KRAS‐addicted lung cancer
Source: EMBO Mol Med. 2019 Mar 4;11(4):e9976. doi: 10.15252/emmm.201809976 (PMC6460353; doi:10.15252/emmm.201809976)
Supplement: Supplementary file 1 — Appendix [file EMMM-11-e9976-s001.pdf]

Appendix

ADAM17 selectively activates the IL-6 trans-signaling/ERK MAPK axis in KRAS-addicted lung cancer

Mohamed I. Saad, Sultan Alhayyani, Louise McLeod, Liang Yu, Mohammad Alanazi, Virginie Deswaerte, Ke Tang, Thierry Jarde, Julian A. Smith, Zdenka Prodanovic, Michelle D. Tate, Jesse J. Balic, D. Neil Watkins, Jason E. Cain, Steven Bozinokski, Elizabeth Algar, Tomohiro Kohmoto, Hiromichi Ebi, Walter Ferlin, Christoph Garbers, Saleela Ruwanpura, Irit Sagi, Stefan Rose-John, Brendan J. Jenkins

Table of contents

Appendix Figure S1. Genetic reduction of ADAM17 expression suppresses the 12-week-old LAC phenotype of *Kras*^{G12D} mice.

Appendix Figure S2. Unaltered expression status of ADAM17 and its regulators in the lungs of *Kras*^{G12D} mice.

Appendix Figure S3. Reduced proliferation, but not apoptosis or angiogenesis, is associated with suppressed tumorigenesis in an A17 pro-treated *KRAS* mutant LAC PDX.

Appendix Table S1. Clinicopathological features of patients used for immunohistochemical staining of pADAM17 and pERK1/2.

Appendix Table S2. Antibody dilutions used for immunoblotting, immunofluorescence and immunohistochemistry.

Appendix Table S3. Primer sequences used for qPCR.

Appendix Table S4. *P* values for differences between experimental groups in figures.

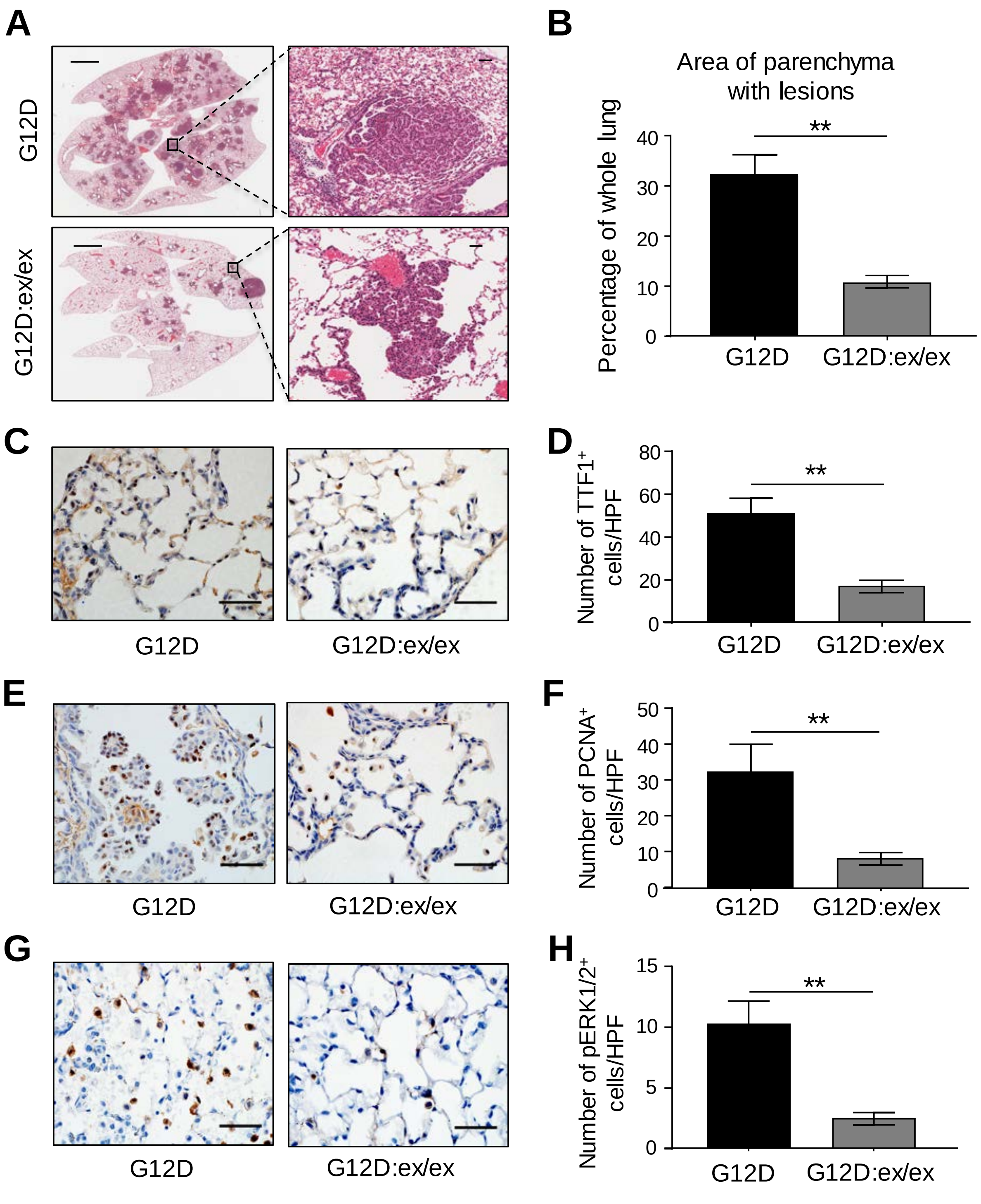

Appendix Figure S1. Genetic reduction of ADAM17 expression suppresses the 12-week-old LAC phenotype of *Kras*^{G12D} mice.

A Representative low power (left) and high power (right) photomicrographs of H&E-stained lung cross-sections from *Kras*^{G12D} and *Kras*^{G12D}:*Adam17*$^{ex/ex}$ mice at 12 weeks following Ad-Cre inhalation. Scale bars, 3mm (left) and 300µm (right).

B Quantification of lung parenchyma area occupied by tumor lesions in the indicated genotypes from (A). Data are presented as the mean ± SEM (n = 6). ***P*<0.01, Student's *t*-test.

C, E, G Representative high power photomicrographs of lung cross-sections from *Kras*^{G12D} and *Kras*^{G12D}:*Adam17*$^{ex/ex}$ mice at 12 weeks following Ad-Cre inhalation that were stained with antibodies against TTF-1 (C), PCNA (E) and pERK1/2 MAPK (G). Scale bars, 100µm.

D, F, H Quantification of positive cells per high power field (HPF) stained for TTF-1 (D), PCNA (F) and pERK1/2 MAPK (H) in lungs of the indicated genotypes from (C), (E) and (G), respectively, at 12 weeks post Ad-Cre inhalation. Data are presented as the mean ± SEM (n = 5). ***P*<0.01, Student's *t*-test.

Exact *P* values are specified in Appendix Table S4.

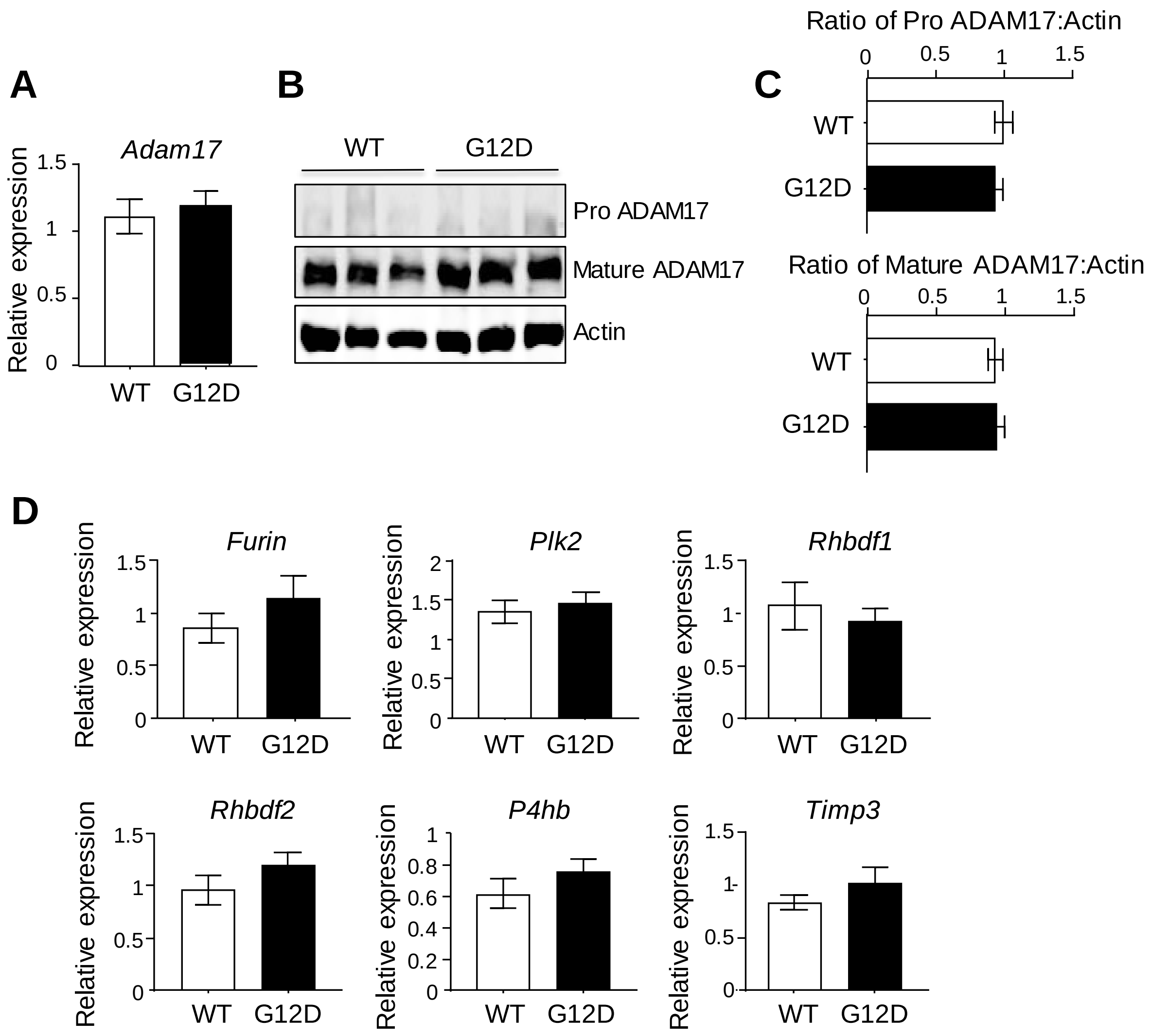

Appendix Figure S2. Unaltered expression status of ADAM17 and its regulators in the lungs of *Kras*^{G12D} mice.

A qPCR gene expression analysis of *Adam17* on lung cDNA from *Kras*^{WT} and *Kras*^{G12D} mice at 6 weeks post inhalations. Expression data are normalized against *18S rRNA*. Data are presented as the mean ± SEM (n = 6 per genotype).

B Immunoblots of representative *Kras*^{WT} and *Kras*^{G12D} lung lysates with antibodies against ADAM17 (detecting pro and mature forms) and Actin. Note that for the Actin blot, lanes 4-6 are reproduced in lanes 1-3 of the Actin blot in Fig 4L.

C Densitometric semi-quantitative analysis of pro and mature ADAM17 protein levels in lung lysates from the mice indicated in (B). Relative expression was determined against Actin. Data are presented as the mean ± SEM (n = 6 per genotype).

D qPCR expression analysis of the indicated genes in lungs of *Kras*^{WT} and *Kras*^{G12D} mice at 6 weeks post inhalations. Expression data are normalized against *18S rRNA*. Data are presented as the mean ± SEM (n = 6 per genotype).

Exact *P* values are specified in Appendix Table S4.

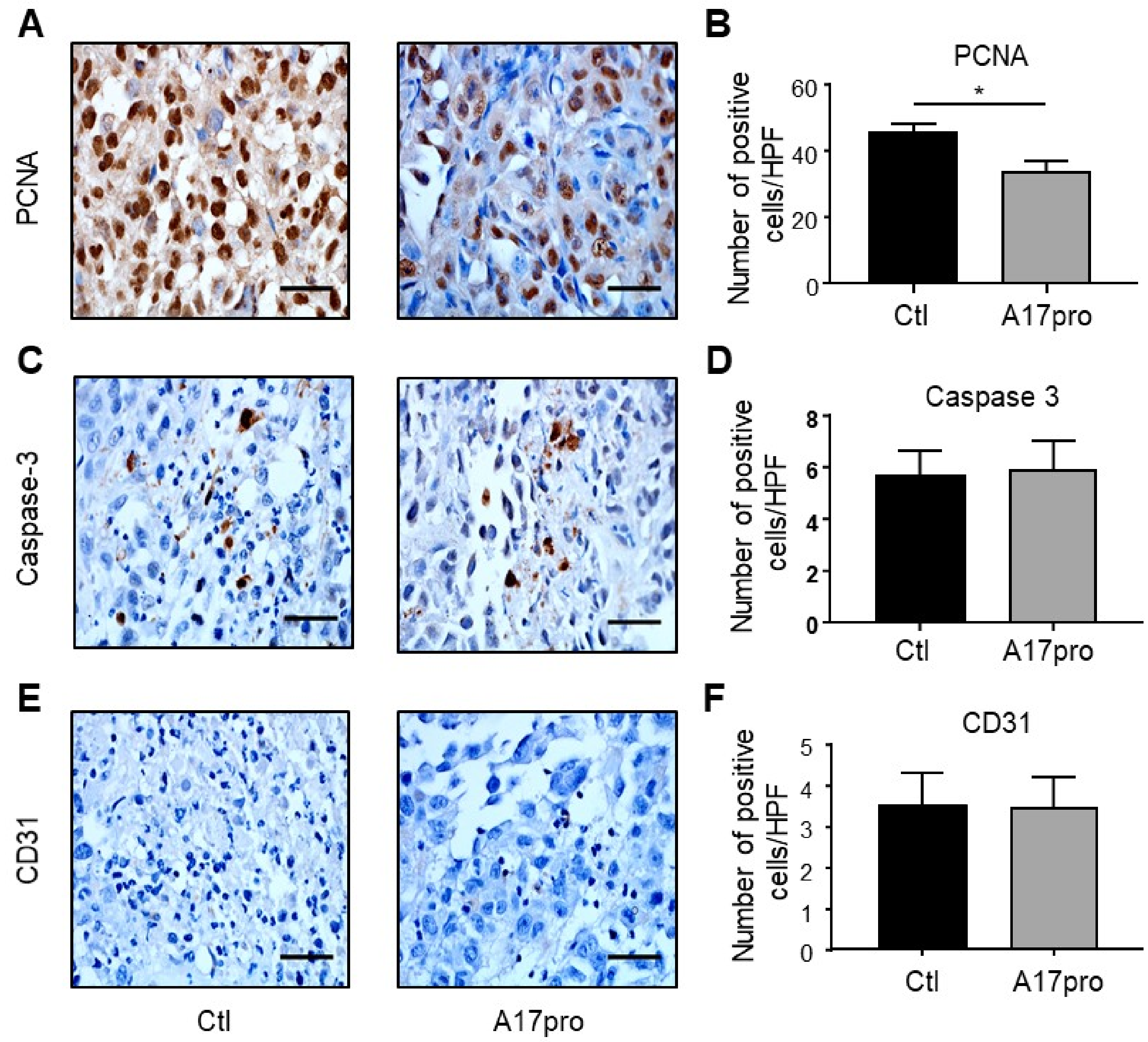

Appendix Figure S3. Reduced proliferation, but not apoptosis or angiogenesis, is associated with suppressed tumorigenesis in an A17 pro-treated *KRAS* mutant LAC PDX.

A, **C**, **E** Representative high power photomicrographs of lung cross-sections from a *KRAS* mutant PDX treated with vehicle control (Ctl) or A17pro (1mg/kg) every second day that were stained with antibodies against PCNA (A), cleaved Caspase-3 (C) and CD31 (E). Scale bars, 100µm.

B, D, F Quantification of positive cells per high power field (HPF) stained for PCNA (B), cleaved Caspase-3 (D) and CD31 (F) in the above treated *KRAS* mutant PDX. Data are presented as the mean ± SEM (n = 5 per genotype). *P<0.05, Student's t-test.

Exact P values are specified in Appendix Table S4.

Appendix Table S1. Clinicopathological features of patients used for immunohistochemical staining of pADAM17 and pERK1/2.

	LAC	Non-LAC
**Mean age**		
Years (range)	69.8 (58-87)	62.0 (47-71)
**Sex [number (%)]**		
Male	10 (55.6)	6 (75)
Female	8 (44.4)	2 (25)
**Smoking history [number (%)]**		
Never	4 (22.2)	2 (25)
Former	10 (55.6)	6 (75)
Current	2 (11.1)	0 (0)
Unknown	2 (11.1)	0 (0)
**Tumor stage[1] [number (%)]**		
I	12 (66.7)	NA
II	4 (22.2)	NA
Unknown	2 (11.1)	NA
***KRAS* status [number (%)]**		
Wild-type	13 (72.2)	NA
Mutant	5 (27.8)	NA

[1]American Joint Committee on Cancer staging for non-small cell lung cancer based on tumor (T)/node (N)/metastasis (M) classification; Stage I (IA/IB), Stage II (IIA/IIB), Stage III (IIIA/IIIB), Stage IV. NA, not applicable.

Appendix Table S2. Antibody dilutions used for immunoblotting, immunofluorescence and immunohistochemistry.

Immunohistochemistry	
**Antibody**	**Dilution**
pThr^{735}-ADAM17	1:100
pThr^{202}/pTyr^{204}-ERK1/2	1:100
pThr^{180}/pTyr^{182}-p38 MAPK	1:100
B220	1:100
CD3	1:100
CD45	1:100
Ly6G	1:100
CD31	1:100
TTF-1	1:100
PCNA	1:5000
Cleaved Caspase-3	1:100
F4/80	1:150
Cleaved Notch1	1:100

Immunofluorescence	
**Antibody**	**Dilution**
ADAM17	1:100
CD31	1:100
pThr^{735}-ADAM17	1:100
CD45	1:100
CC10	1:100
SP-C	1:100
IL-6R	1:100
TGFα	1:100

Nrg1	1:100
EGF	1:100
Podoplanin	1:100
Alexa Fluor secondary antibodies	1:200
**Immunoblotting**	
**Antibody**	**Dilution**
ADAM17	1:500
pThr^{735}-ADAM17	1:500
Myc	1:1000
pTyr^{705}-STAT3	1:1000
pSer^{727}-STAT3	1:1000
STAT3	1:1000
pSer^{473}-AKT	1:1000
AKT	1:1000
pThr^{202}/pTyr^{204}-ERK1/2	1:1000
ERK1/2	1:1000
pThr^{180}/pTyr^{182}-p38 MAPK	1:1000
p38 MAPK	1:1000
pTyr^{1068}-EGFR	1:1000
EGFR	1:1000
pSer^{82}-HSP27	1:1000
Cleaved Notch1	1:500
IL-6R	1:500
Nrg1	1:1000
pTyr^{1289}-ErbB3	1:1000
ErbB3	1:1000
TGFα	1:500

Actin	1:500

Appendix Table S3. Primer sequences used for qPCR.

Gene	Forward	Reverse
Ccnd1	AGTGCGTGCAGAAGGAGATT	AGGAAGCGGTCCAGGTAGTT
Ccnb1	TAGGGTGTCTTCTCGAATCGGGG	ACGCTTGGCGCCTGCCATA
Cdc42	GAAAATGTGAAAGAAAAGTGG	TCTGGAGTAATAGGCTTCTGT
Myc	CGGACACACAACGTCTTGGAA	AGGATGTAGGCGGTGGCTTTT
Il1b	CAACCAACAAGTGATATTCTCCATG	GATCCACACTCTCCAGCTGCA
Il7a	ACCGCAATGAAGACCCTGAT	TCCCTCCGCATTGACACA
Il6	ATGGATGCTACCAAACTGGAT	TGAAGGACTCTGGCTTTGTCT
Cxcl2	AACATCCAGAGCTTGAGTGTGA	TTCAGGGTCAAGGCAAACTT
Vegf	GCACACAGGACGGCTTGAAGA	CCCACGACAGAAGAGCAGA
Mmp2	GCTCTATGGGCCCTCCCCCG	CGTGGTGTCACTGTCCGCCA
Il6r	AAGCAGCAGGCAATGTTACC	CATAAATAGTCCCCAGTGTCG
Hes1	ACCTTCCAGTGGCTCCTC	TTTAGTGTCCGTCAGAAGAGAG
Hey1	GCCGAAGTTGCCCGTTATCTG	TGTGTGGGTGATGTCCGAAGG
Furin	GGCTCTAAGAAAGGGGGTTG	TGCAAAAGGCACATCAGAAG
Plk2	CGGTGCTGAAATACTTTTCTCAT	TCGGGAGATCACCACCAT
Rhbdf1	AGCTATGTCAAGCGGCAGAT	TTCCGAAGTACCGAGTCCAC
Rhbdf2	TGCCTGACGATGTCTTTGAG	TAGCTTCGGTTGAGCCAGTT
P4hb	TGACCTGGCTCAGCAGTATG	AAACCGATGACCGTCACTTC
Timp3	TCCTAGACCCAGTTCCATATACACTTC	TTGGACTTCTGCCAACTTCCTT

Appendix Table S4. *P* values for differences between experimental groups in figures.

		**Figure 1B**		
**Student's *t*-test**		**Significant?**	**Summary**	***P* value**
G12D	G12D:ex/+	Yes	*	0.0347
G12D	G12D:ex/ex	Yes	***	0.0001
G12D:ex/+	G12D:ex/ex	Yes	***	0.0001
		**Figure 1C**		
**Student's *t*-test**		**Significant?**	**Summary**	***P* value**
G12D	G12D:ex/+	No	ns	0.1236
G12D	G12D:ex/ex	Yes	***	0.0001
G12D:ex/+	G12D:ex/ex	Yes	*	0.0260
		**Figure 1E**		
**Student's *t*-test**		**Significant?**	**Summary**	***P* value**
WT	G12D	Yes	**	0.0022
WT	G12D:ex/+	Yes	**	0.0022
WT	G12D:ex/ex	No	ns	0.3939
G12D	G12D:ex/+	Yes	**	0.0043
G12D	G12D:ex/ex	Yes	**	0.0022
G12D:ex/+	G12D:ex/ex	Yes	*	0.0152
		**Figure 2B**		
**Student's *t*-test**		**Significant?**	**Summary**	***P* value**
WT	G12D	Yes	**	0.0022
WT	G12D:ex/ex	No	ns	0.2403
G12D	G12D:ex/ex	Yes	**	0.0022
		**Figure 2C**		
**Student's *t*-test**		**Significant?**	**Summary**	***P* value**
		Ccnd1		
WT	G12D	Yes	*	0.0181
WT	G12D:ex/ex	No	ns	0.3374
G12D	G12D:ex/ex	Yes	*	0.0428
		Ccnb1		
WT	G12D	Yes	*	0.0110
WT	G12D:ex/ex	No	ns	0.0635
G12D	G12D:ex/ex	Yes	**	0.0034
		Cdc42		
WT	G12D	Yes	*	0.0124
WT	G12D:ex/ex	No	ns	0.4533
G12D	G12D:ex/ex	Yes	*	0.0462
		Myc		
WT	G12D	Yes	*	0.0181
WT	G12D:ex/ex	No	ns	0.4026
G12D	G12D:ex/ex	Yes	*	0.0290
		**Figure 2E**		
**Student's *t*-test**		**Significant?**	**Summary**	***P* value**
G12D	G12D:ex/ex	Yes	**	0.0079
		**Figure 2G**		
**Student's *t*-test**		**Significant?**	**Summary**	***P* value**
WT	G12D	Yes	**	0.0022
WT	G12D:ex/ex	Yes	**	0.0022
G12D	G12D:ex/ex	Yes	**	0.0022
		**Figure 3H**		
**Student's *t*-test**		**Significant?**	**Summary**	***P* value**
Kras^{Kras}	Kras$^{Kras:ex/ex}$	No	ns	0.8502

		**Figure 4B**		
**Student's *t*-test**		**Significant?**	**Summary**	***P* value**
WT	G12D	Yes	**	0.0079
		**Figure 4D**		
**Student's *t*-test**		**Significant?**	**Summary**	***P* value**
WT	G12D	Yes	*	0.0317
		**Figure 4F**		
**Student's *t*-test**		**Significant?**	**Summary**	***P* value**
WT	G12D	Yes	**	0.0079
WT	G12D	No	ns	0.8413
		**Figure 4H**		
**Student's *t*-test**		**Significant?**	**Summary**	***P* value**
Ctl	SB	Yes	**	0.0079
Ctl	U0	No	ns	>0.9999
SB	U0	Yes	*	0.0317
		**Figure 4J**		
**Student's *t*-test**		**Significant?**	**Summary**	***P* value**
Ctl	SB	Yes	**	0.0079
Ctl	SB	Yes	*	0.0317
		**Figure 4K**		
**Student's *t*-test**		**Significant?**	**Summary**	***P* value**
Ctl	SB	Yes	**	0.0012
Ctl	SB	Yes	**	0.0095
		**Figure 4N**		
**Student's *t*-test**		**Significant?**	**Summary**	***P* value**
G12D	G12D:ex/ex	Yes	**	0.0079
		**Figure 5A**		
**Student's *t*-test**		**Significant?**	**Summary**	***P* value**
		**sIL-6R**		
WT	G12D	Yes	*	0.0334
WT	G12D:ex/ex	No	ns	0.3577
G12D	G12D:ex/ex	Yes	**	0.0016
		**TNFα**		
WT	G12D	No	ns	0.8481
WT	G12D:ex/ex	No	ns	0.4436
G12D	G12D:ex/ex	No	ns	0.4931
		**Figure 5E**		
**Student's *t*-test**		**Significant?**	**Summary**	***P* value**
Ctl	1F7	Yes	*	0.0357
Ctl	25F10	No	ns	0.1429
1F7	25F10	No	ns	0.1000
		**Figure 6B**		
**Student's *t*-test**		**Significant?**	**Summary**	***P* value**
Norm	LAC	Yes	*	0.0228
		**Figure 6C**		
**Student's *t*-test**		**Significant?**	**Summary**	***P* value**
WT	MUT	Yes	*	0.0228
WT	MUT	Yes	**	0.0056
		**Figure 6H**		
**Student's *t*-test**		**Significant?**	**Summary**	***P* value**
HBEC	A549	Yes	**	0.0012
HBEC	H23	Yes	**	0.0043
HBEC	H2228	Yes	**	0.0012
A549	H23	No	ns	0.1490

A549	H2228	Yes	**	0.0012
H23	H2228	Yes	**	0.0025
**Figure 6I**				
**Student's *t*-test**		**Significant?**	**Summary**	***P* value**
Ctl	KO	Yes	**	0.0043
**Figure 6J**				
**Student's *t*-test**		**Significant?**	**Summary**	***P* value**
Ctl	KO	Yes	*	0.0286
**Figure 6K**				
**Student's *t*-test**		**Significant?**	**Summary**	***P* value**
Ctl	KO	Yes	***	0.0006
**Figure 6L**				
**Multiple Student's *t*-test**		**Significant?**	**Summary**	***P* value**
(0) Ctl	KO	No	ns	0.2838
(3) Ctl	KO	No	ns	0.3088
(6) Ctl	KO	Yes	*	0.0475
(9) Ctl	KO	Yes	*	0.0246
(12) Ctl	KO	Yes	*	0.0320
(15) Ctl	KO	Yes	*	0.0445
(18) Ctl	KO	Yes	*	0.0180
(21) Ctl	KO	Yes	*	0.0256
**Figure 6M**				
**Student's *t*-test**		**Significant?**	**Summary**	***P* value**
Ctl	KO	Yes	**	0.0079
**Figure 7B**				
**Student's *t*-test**		**Significant?**	**Summary**	***P* value**
Ctl	A17pro	Yes	**	0.0057
**Figure 7D**				
**Student's *t*-test**		**Significant?**	**Summary**	***P* value**
Ctl	A17pro	Yes	**	0.0072
**Figure 7F**				
**Student's *t*-test**		**Significant?**	**Summary**	***P* value**
Ctl	A17pro	Yes	**	0.0022
**Figure 7H**				
**Student's *t*-test**		**Significant?**	**Summary**	***P* value**
Ctl	A17pro	Yes	*	0.0481
**Figure 7I**				
**Student's *t*-test**		**Significant?**	**Summary**	***P* value**
Ctl	A17pro	Yes	*	0.0411
**Figure 7J**				
**Multiple Student's *t*-test**		**Significant?**	**Summary**	***P* value**
(0) Ctl	A17pro	No	ns	0.6895
(2) Ctl	A17pro	No	ns	0.2255
(4) Ctl	A17pro	No	ns	0.4237
(6) Ctl	A17pro	Yes	*	0.0065
(8) Ctl	A17pro	Yes	*	0.0017
(10) Ctl	A17pro	Yes	*	0.0069
(12) Ctl	A17pro	Yes	*	0.0065
(14) Ctl	A17pro	Yes	*	0.0062
**Figure 7K**				
**Student's *t*-test**		**Significant?**	**Summary**	***P* value**
Ctl	A17pro	Yes	*	0.0411
**Figure 7M**				
**Student's *t*-test**		**Significant?**	**Summary**	***P* value**

Ctl	A17pro	Yes	*	0.0240

Figure EV1A

**Student's *t*-test**		**Significant?**	**Summary**	***P* value**
G12D	G12D:ex/ex	Yes	**	0.0022

Figure EV1C

**Student's *t*-test**		**Significant?**	**Summary**	***P* value**
G12D	G12D:ex/ex	Yes	*	0.0152

Figure EV1D

**Student's *t*-test**		**Significant?**	**Summary**	***P* value**
G12D	G12D:ex/ex	Yes	**	0.0043

Figure EV1E

**Student's *t*-test**		**Significant?**	**Summary**	***P* value**
G12D	G12D:ex/ex	Yes	*	0.0433

Figure EV1F

**Student's *t*-test**		**Significant?**	**Summary**	***P* value**
G12D	G12D:ex/ex	Yes	*	0.0455

Figure EV2B

**Student's *t*-test**		**Significant?**	**Summary**	***P* value**
WT	G12D	Yes	**	0.0022
WT	G12D:ex/ex	Yes	**	0.0043
G12D	G12D:ex/ex	Yes	*	0.0411

Figure EV2D

**Student's *t*-test**		**Significant?**	**Summary**	***P* value**
WT	G12D	Yes	**	0.0022
WT	G12D:ex/ex	Yes	*	0.0411
G12D	G12D:ex/ex	No	ns	0.0931

Figure EV2F

**Student's *t*-test**		**Significant?**	**Summary**	***P* value**
WT	G12D	Yes	*	0.0260
WT	G12D:ex/ex	No	ns	0.5887
G12D	G12D:ex/ex	Yes	*	0.0152

Figure EV2H

**Student's *t*-test**		**Significant?**	**Summary**	***P* value**
WT	G12D	No	ns	0.1667
WT	G12D:ex/ex	No	ns	0.9069
G12D	G12D:ex/ex	No	ns	0.2900

Figure EV2I

Il1b

**Student's *t*-test**		**Significant?**	**Summary**	***P* value**
G12D	G12D:ex/ex	Yes	**	0.0032

Il17a

**Student's *t*-test**		**Significant?**	**Summary**	***P* value**
G12D	G12D:ex/ex	Yes	*	0.0353

Il6

**Student's *t*-test**		**Significant?**	**Summary**	***P* value**
G12D	G12D:ex/ex	Yes	*	0.0489

Figure EV2K

**Student's *t*-test**		**Significant?**	**Summary**	***P* value**
G12D	G12D:ex/ex	No	ns	0.3651

Figure EV2M

**Student's *t*-test**		**Significant?**	**Summary**	***P* value**
G12D	G12D:ex/ex	No	ns	0.1688

Figure EV2N

Cxcl2

Student's *t*-test		Significant?	Summary	*P* value
G12D	G12D:ex/ex	No	ns	0.1775
		Vegf		
**Student's *t*-test**		**Significant?**	**Summary**	***P* value**
G12D	G12D:ex/ex	No	ns	0.5887
		Mmp2		
**Student's *t*-test**		**Significant?**	**Summary**	***P* value**
G12D	G12D:ex/ex	No	ns	0.6623
		**Figure EV3C**		
**Student's *t*-test**		**Significant?**	**Summary**	***P* value**
G12D	G12D:ex/ex	No	ns	0.0649
		**Figure EV3D**		
		Hes1		
**Student's *t*-test**		**Significant?**	**Summary**	***P* value**
G12D	G12D:ex/ex	No	ns	0.1797
		Hey1		
**Student's *t*-test**		**Significant?**	**Summary**	***P* value**
G12D	G12D:ex/ex	No	ns	0.3095
		**Figure EV5A**		
**Student's *t*-test**		**Significant?**	**Summary**	***P* value**
Ctl	KO	Yes	**	0.0012
		**Figure EV5B**		
**Student's *t*-test**		**Significant?**	**Summary**	***P* value**
Ctl	KO	Yes	**	0.0043
		**Figure EV5C**		
**Student's *t*-test**		**Significant?**	**Summary**	***P* value**
Ctl	KO	Yes	**	0.0022
		**Figure EV5D**		
**Student's *t*-test**		**Significant?**	**Summary**	***P* value**
Ctl	A17pro	Yes	**	0.0022
Ctl	A17pro	Yes	***	0.0003
		**Figure EV5E**		
**Student's *t*-test**		**Significant?**	**Summary**	***P* value**
Ctl	A17pro	Yes	*	0.0254
Ctl	A17pro	Yes	***	0.0007
		**Figure EV5F**		
**Student's *t*-test**		**Significant?**	**Summary**	***P* value**
Ctl	A17pro	Yes	*	0.0424
Ctl	A17pro	Yes	**	0.0022
		**Figure EV5H**		
**Student's *t*-test**		**Significant?**	**Summary**	***P* value**
Ctl	KO	Yes	*	0.0286
Ctl	GW	Yes	*	0.0286
Ctl	A17pro	Yes	*	0.0286
KO	GW	No	ns	0.2739
KO	A17pro	No	ns	0.7490
GW	A17pro	No	ns	0.1402
		**Figure EV5I**		
**Student's *t*-test**		**Significant?**	**Summary**	***P* value**
Ctl	GW	Yes	*	0.0496
Ctl	GW	Yes	*	0.0295
		**Figure EV5J**		
**Student's *t*-test**		**Significant?**	**Summary**	***P* value**
Ctl	GW	Yes	*	0.0466

Ctl	GW	Yes	*	0.0357

Figure EV5K

Student's *t*-test		Significant?	Summary	*P* value
Ctl	GW	Yes	**	0.0079
Ctl	GW	Yes	**	0.0079

Figure S1B

Student's *t*-test		Significant?	Summary	*P* value
G12D	G12D:ex/ex	Yes	**	0.0022

Figure S1D

Student's *t*-test		Significant?	Summary	*P* value
G12D	G12D:ex/ex	Yes	**	0.0022

Figure S1F

Student's *t*-test		Significant?	Summary	*P* value
G12D	G12D:ex/ex	Yes	**	0.0079

Figure S1H

Student's *t*-test		Significant?	Summary	*P* value
G12D	G12D:ex/ex	Yes	**	0.0079

Figure S2A

Student's *t*-test		Significant?	Summary	*P* value
WT	G12D	No	ns	0.7221

Figure S2C

Student's *t*-test		Significant?	Summary	*P* value
WT	G12D	No	ns	0.2046
WT	G12D	No	ns	0.9372

Figure S2D

Furin

Student's *t*-test		Significant?	Summary	*P* value
WT	G12D	No	ns	0.5476

Plk2

Student's *t*-test		Significant?	Summary	*P* value
WT	G12D	No	ns	0.8413

Rhbdf1

Student's *t*-test		Significant?	Summary	*P* value
WT	G12D	No	ns	>0.9999

Rhbdf2

Student's *t*-test		Significant?	Summary	*P* value
WT	G12D	No	ns	0.4206

P4hb

Student's *t*-test		Significant?	Summary	*P* value
WT	G12D	No	ns	0.2783

Timp3

Student's *t*-test		Significant?	Summary	*P* value
WT	G12D	No	ns	0.3326

Figure S3B

Student's *t*-test		Significant?	Summary	*P* value
Ctl	A17pro	Yes	*	0.0133

Figure S3D

Student's *t*-test		Significant?	Summary	*P* value
Ctl	A17pro	No	ns	0.8468

Figure S3F

Student's *t*-test		Significant?	Summary	*P* value
Ctl	A17pro	No	ns	0.9496
