# Supplementary material for: ADAM17 selectively activates the IL‐6 trans‐signaling/ERK MAPK axis in KRAS‐addicted lung cancer
Source: EMBO Mol Med. 2019 Mar 4;11(4):e9976. doi: 10.15252/emmm.201809976 (PMC6460353; doi:10.15252/emmm.201809976)
Supplement: Supplementary file 2 — Expanded View Figures PDF [file EMMM-11-e9976-s002.pdf]

Expanded View Figures

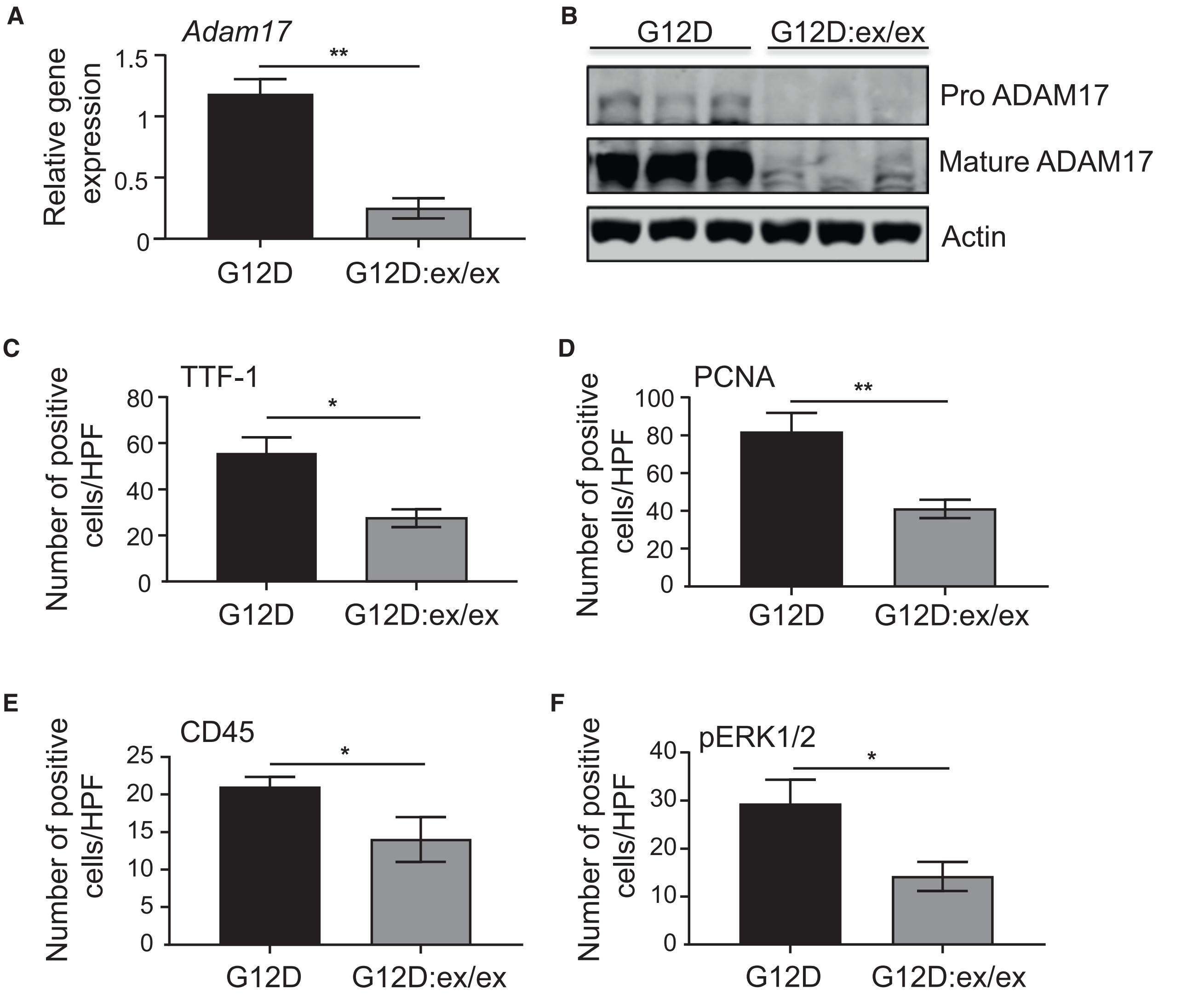

Figure EV1. Reduced immunohistochemical staining of cellular markers in lung lesions associated with LAC upon genetic reduction in ADAM17 expression in *Kras*^{G12D} mice.

A qPCR gene expression analysis of *Adam17* on lung cDNA from *Kras*^{G12D} and *Kras*^{G12D}:*Adam17*$^{ex/ex}$ mice at 6 weeks following Ad-Cre inhalation (n = 6 per group). Expression data are normalized against *18S rRNA*. **P < 0.01, Student's t-test, mean ± SEM.

B Immunoblots of representative *Kras*^{G12D} and *Kras*^{G12D}:*Adam17*$^{ex/ex}$ lung lysates with antibodies against total (pro and mature) ADAM17 and actin.

C–F Quantification of positive cells per high-power field (HPF) stained for TTF-1 (C), PCNA (D), CD45 (E), and pERK1/2 MAPK (F) in lung lesions of the indicated genotypes (n = 6 per genotype) at 6 weeks post-Ad-Cre inhalation. *P < 0.05, **P < 0.01, Student's t-test, mean ± SEM.

Data information: Exact P values are specified in Appendix Table S4.

 © 2019 The Authors

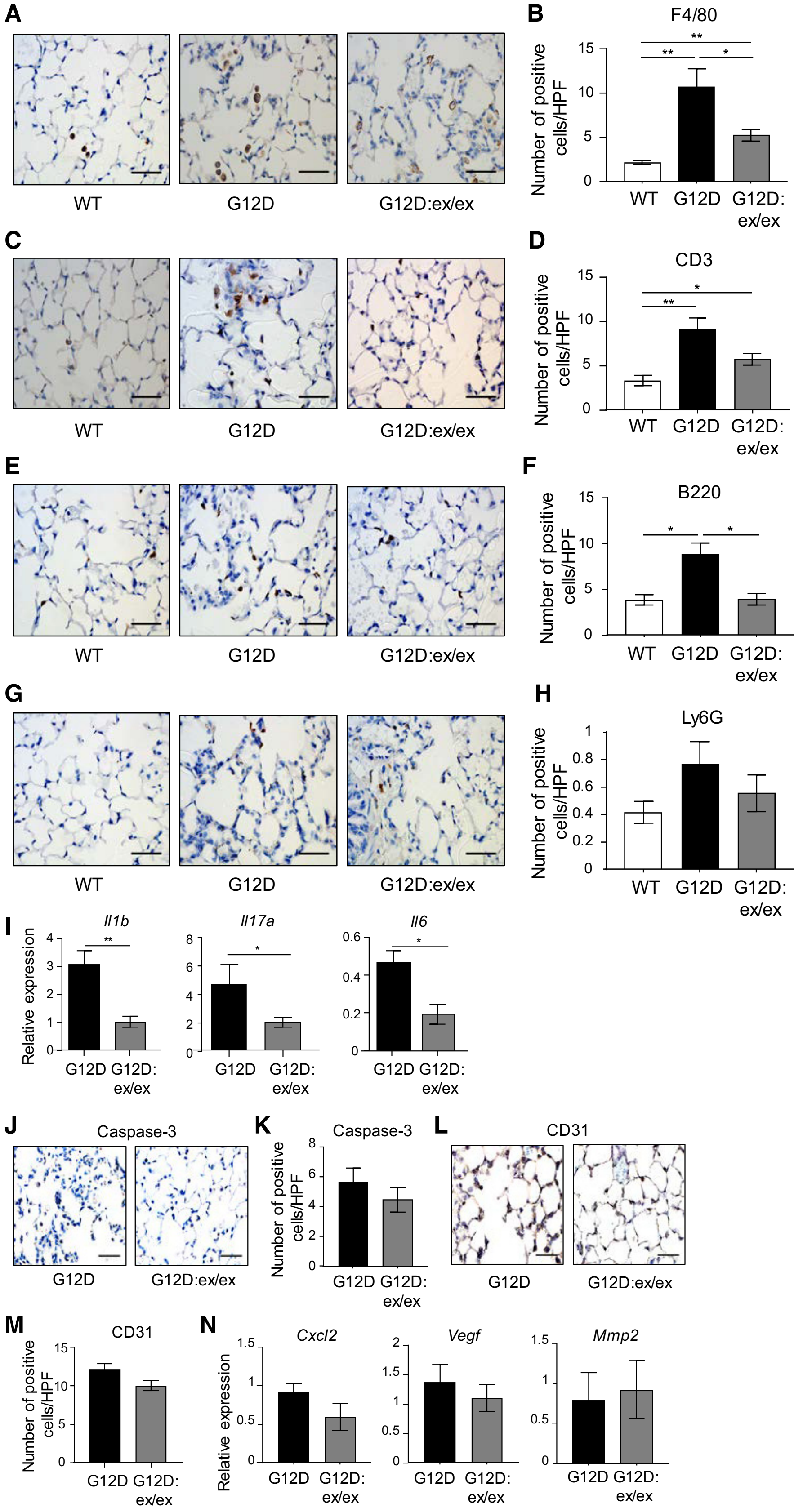

Figure EV2. Reduced inflammatory infiltrates, but not apoptosis or angiogenesis, is associated with suppressed tumorigenesis in the lungs of *Kras*^{G12D}:*Adam17*$^{ex/ex}$ mice.

A–N (A, C, E, G, J, L) Representative high-power photomicrographs of lung cross-sections from *Kras*^{WT}, *Kras*^{G12D}, and *Kras*^{G12D}:*Adam17*$^{ex/ex}$ mice at 6 weeks following inhalations that were stained with antibodies against F4/80 (A), CD3 (C), B220 (E), Ly6G (G), cleaved Caspase-3 (J), and CD31 (L). Scale bars, 100 μm. (B, D, F, H, K, M) Quantification of positive cells per high-power field (HPF) stained for F4/80 (B), CD3 (D), B220 (F), Ly6G (H), cleaved Caspase-3 (K), and CD31 (M) in lungs of the indicated genotypes (n = 6 per genotype) from (A), (C), (E), (G), (J), and (L), respectively. *P < 0.05, **P < 0.01, Student's t-test, mean ± SEM. (I, N) qPCR expression analyses of representative inflammatory (I) and angiogenic (N) genes on lung cDNA from the indicated genotypes (n = 6 per genotype). Expression data are normalized against *18S rRNA*. *P < 0.05, **P < 0.01, Student's t-test, mean ± SEM.

Data information: Exact P values are specified in Appendix Table S4.

© 2019 The Authors

Figure EV3. Expression analysis of the ADAM17 substrate, IL-6R, in distinct cellular compartments within the lungs of *Kras*^{G12D} mice.

A, B Immunoblots of representative individual lysates from liver and skeletal muscle (A), as well as lung (B), of *Kras*^{G12D} and *Kras*^{G12D}:*Adam17*$^{ex/ex}$ mice, at 6 weeks following Ad-Cre inhalation, with the indicated antibodies.

C, D qPCR expression analysis of the *Il6r* gene (C), and the indicated Notch1-regulated *Hes1* and *Hey1* genes (D), in lungs of *Kras*^{G12D} and *Kras*^{G12D}:*Adam17*$^{ex/ex}$ mice (n = 6 per genotype) at 6 weeks following inhalations. Expression data are normalized against *18S rRNA*, mean ± SEM.

E–H Immunofluorescence analyses of lung cross-sections of *Kras*^{G12D} mice showing co-localization of staining with an anti-IL-6R antibody and antibodies for club cells (CC10), alveolar type II cells (SPC), endothelial cells (CD31), and immune cells (CD45). DAPI (blue) is used for nuclear staining. Images are representative of three mice. Scale bars, 100 μm.

Data information: Exact *P* values are specified in Appendix Table S4.

© 2019 The Authors

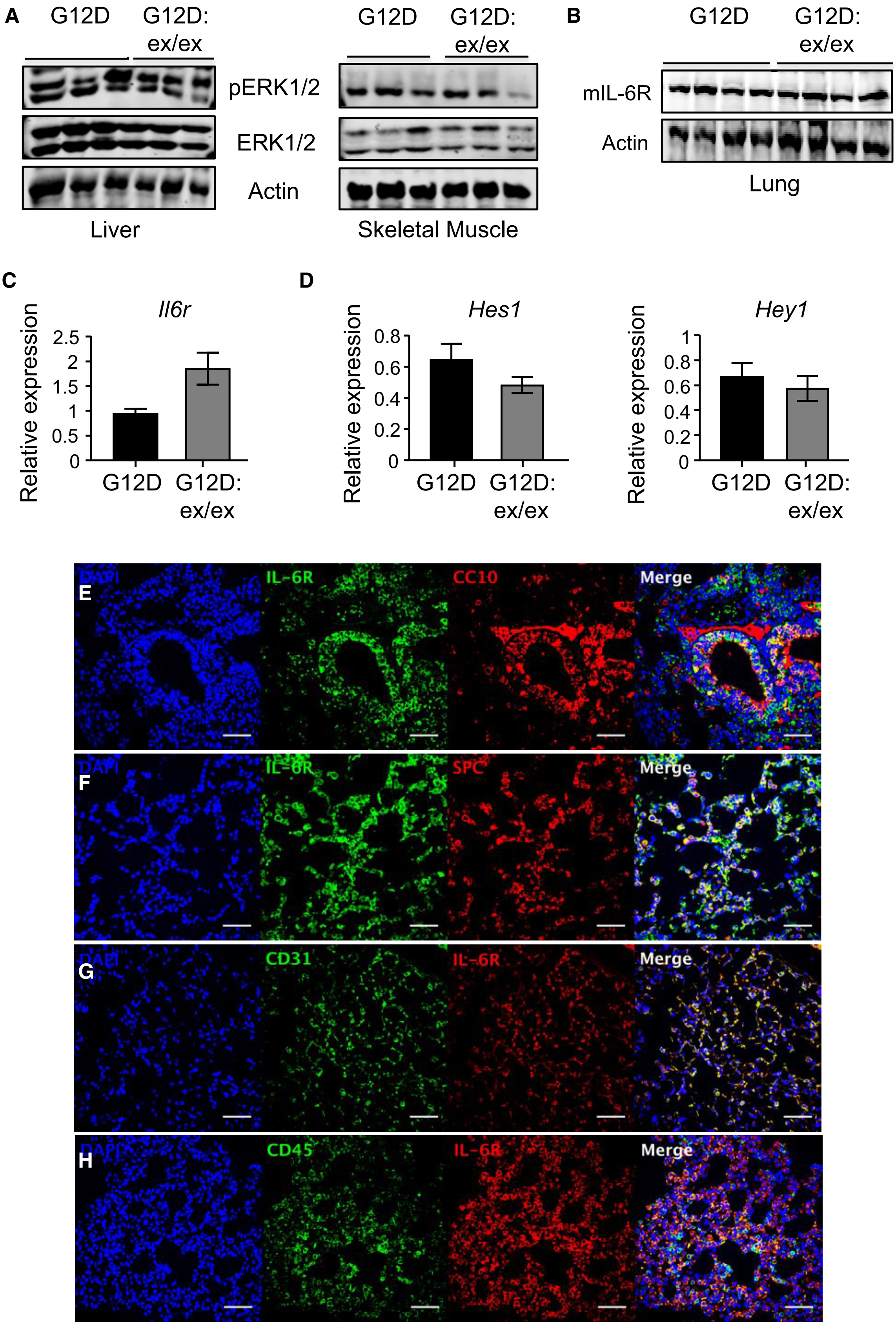
A
G12D
G12D: ex/ex
pERK1/2
ERK1/2
Actin
Liver
G12D
G12D: ex/ex
Skeletal Muscle
B
G12D
G12D: ex/ex
mIL-6R
Actin
Lung
C
Il6r
Relative expression
2.5
2
1.5
1
0.5
0
G12D
G12D: ex/ex
D
Hes1
Relative expression
0.8
0.6
0.4
0.2
0
G12D
G12D: ex/ex
Hey1
Relative expression
1
0.8
0.6
0.4
0.2
0
G12D
G12D: ex/ex
E
DAPI
IL-6R
CC10
Merge
F
DAPI
IL-6R
SPC
Merge
G
DAPI
CD31
IL-6R
Merge
H
DAPI
CD45
IL-6R
Merge

Figure EV3.

Figure EV4. ADAM17 expression analysis in human LAC patients and cell lines and CRISPR/Cas9 gene ablation of ADAM17 in human cell lines.

A Immunofluorescence analyses of human LAC tissue showing $pThr^{735}$-ADAM17 (pA17) expression along with SPC or CD45 expression. DAPI (blue) is used for nuclear staining. Images are representative of three patients. Scale bars, 50 μm. White arrows point to dual-stained (A17, SPC) positive cells.

B *ADAM17* mRNA expression levels in LAC patients obtained from The Cancer Genome Atlas (TCGA) Research Network. Left graph: No statistical difference in *ADAM17* expression was observed between paired tumor (T) and non-tumor (NT) LAC patient samples (n = 57). P = 0.252, Student's t-test. Right graph: No statistical difference in *ADAM17* expression was observed between wild-type *KRAS* (WT, n = 438) and mutant *KRAS* (MUT, n = 75) LAC patient tumors. P = 0.354, Student's t-test. In both graphs, the value in parenthesis indicates the mean of *ADAM17* mRNA expression level. Each box spans the lower (bottom line of box) and upper (top line of box) quartiles of ADAM17 mRNA levels. The line across each box represents the median expression value. The whiskers represent the highest and lowest data points for ADAM17 mRNA levels.

C Immunoblots of representative individual patient lung lysates from normal (cancer-free) and LAC tumor tissue biopsies with antibodies against the indicated targets.

D Immunofluorescence analyses of cultured normal HBECs and LAC cell lines A549, NIH-H23, and NIH-H2228 showing pA17 expression. Images are representative of three independent experiments. Scale bars, 10 μm.

E CRISPR/Cas9 strategy for creating ADAM17 knockout (KO) A549 and NIH-H23 cell lines.

F Immunoblots of representative lysates from control and KO A549 and NIH-H23 cells with antibodies against total ADAM17 (detecting pro- and mature forms) and actin.

© 2019 The Authors

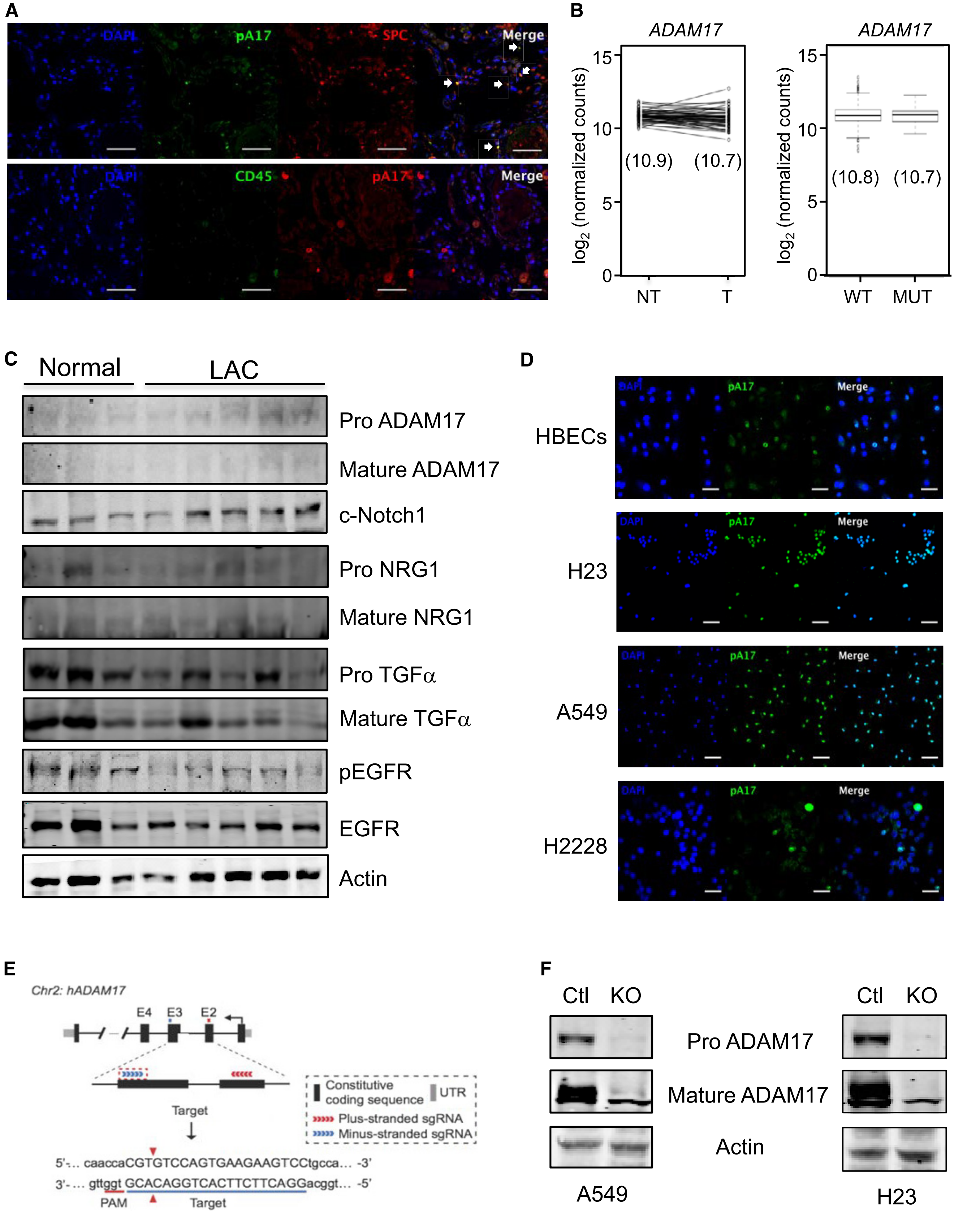
A
DAPI
pA17
SPC
Merge
DAPI
CD45
pA17
Merge
B
ADAM17
15
10
5
0
log2 (normalized counts)
(10.9)
(10.7)
NT
T
ADAM17
(10.8)
(10.7)
WT
MUT
C
Normal
LAC
Pro ADAM17
Mature ADAM17
c-Notch1
Pro NRG1
Mature NRG1
Pro TGFα
Mature TGFα
pEGFR
EGFR
Actin
D
HBECs
H23
A549
H2228
DAPI
pA17
Merge
E
Chr2: hADAM17
E4
E3
E2
Target
Constitutive coding sequence
UTR
Plus-stranded sgRNA
Minus-stranded sgRNA
5'-... caaccaCGTGTCCAGTGAAGAAGTCCtgcca... -3'
3'-... gttggt GCACAGGTCACTTCTTCAGGacggt... -5'
PAM
Target
F
Ctl
KO
Pro ADAM17
Mature ADAM17
Actin
A549
H23

Figure EV4.

Figure EV5. Genetic and inhibitor targeting of ADAM17 in *KRAS* mutant human LAC cell lines suppresses cell viability, spheroid formation, and proliferation *in vitro*.

A, B ATP proliferation (A) and MTT viability (B) assays of NIH-H23 cells transduced with non-targeted control sgRNA (Ctl) and ADAM17 sgRNA (knockout, KO). Data are from three independent experiments performed in triplicate. $^{**}P < 0.01$, Student's t-test, mean ± SEM.

C ELISA of sIL-6R protein levels in culture supernatants from matching NIH-H23 cells in (A) and (B) cultured over 24 h. Data are from 3 independent experiments. $^{**}P < 0.01$, Student's t-test, mean ± SEM.

D, E Proliferation (D) and viability (E) of human A549 and NIH-H23 LAC cells treated with the specific ADAM17 inhibitor A17pro (2 μM) for 24 h and assessed by ATP (D) and MTT (E) assays. Data are combined from at least three independent experiments (performed in triplicate). $^{*}P < 0.05$, $^{**}P < 0.01$, $^{***}P < 0.001$, Student's t-test, mean ± SEM.

F ELISA of sIL-6R protein concentrations in cell culture supernatants of A549 and NIH-H23 cells treated as above with A17pro. Data are combined from at least three independent experiments. $^{*}P < 0.05$, $^{**}P < 0.01$, Student's t-test, mean ± SEM.

G Shown are representative images of the indicated A549 cell spheroid aggregates formed over 3–5 days from three independent experiments comprising biological replicates. A549 control (non-targeted) cells were also treated with the dual ADAM10/17 inhibitor, GW280264X (2 mM), or A17pro (2 μM). Scale bars, 100 μm.

H Graph depicts quantification of the average size (presented in arbitrary units) of spheroid aggregates as measured from at least 20 individual aggregates per cell group from (G). Data are presented from three independent experiments as the mean ± SEM. $^{*}P < 0.05$, Student's t-test, mean ± SEM.

I, J Proliferation (I) and viability (J) of human A549 and NIH-H23 LAC cells treated with GW280264X (2 μM) for 24 h and assessed by ATP (I) and MTT (J) assays. Data are combined from at least three independent experiments (performed in triplicate). $^{*}P < 0.05$, Student's t-test, mean ± SEM.

K ELISA of sIL-6R protein concentrations in cell culture supernatants of A549 and NIH-H23 cells treated as above with GW280264X. Data are combined from at least three independent experiments. $^{**}P < 0.01$, Student's t-test, mean ± SEM.

Data information: Exact P values are specified in Appendix Table S4.

© 2019 The Authors

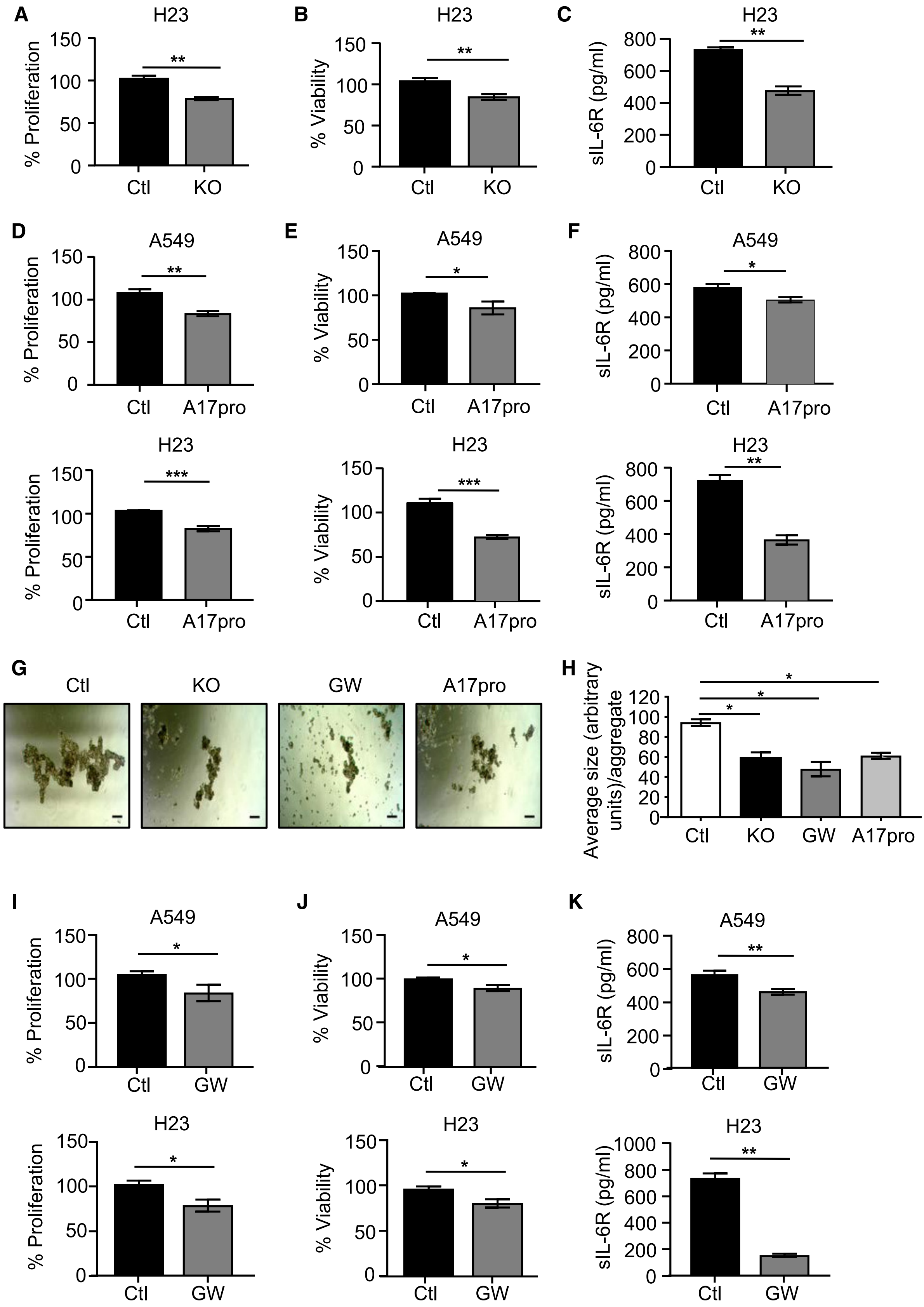
A
H23
% Proliferation
150
100
50
0
**
Ctl
KO
B
H23
% Viability
C
H23
sIL-6R (pg/ml)
800
600
400
200
D
A549
A17pro
E
*
F
***
G
Ctl
KO
GW
A17pro
H
Average size (arbitrary units)/aggregate
120
80
60
40
20
I
J
K
1000

Figure EV5.
