# Supplementary figures and images for: ADAM17 selectively activates the IL‐6 trans‐signaling/ERK MAPK axis in KRAS‐addicted lung cancer
Source: EMBO Mol Med. 2019 Mar 4;11(4):e9976. doi: 10.15252/emmm.201809976 (PMC6460353; doi:10.15252/emmm.201809976)

**Figure S2**

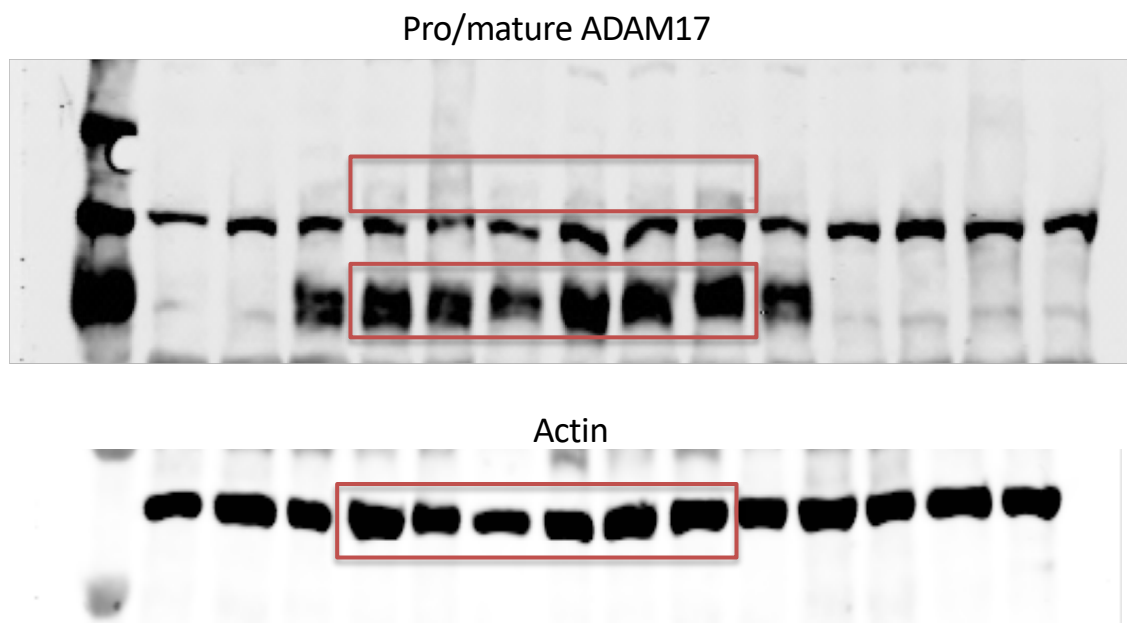

The lanes 7-14 from the left in the actin blot are reproduced and used in Fig. 4L.

Supplement: Supplementary file 3 — Source Data for Expanded View [file EMMM-11-e9976-s008.zip › emmm201809976-sup-0008-SDataFigS2.pdf]

Figure 2D

Myc

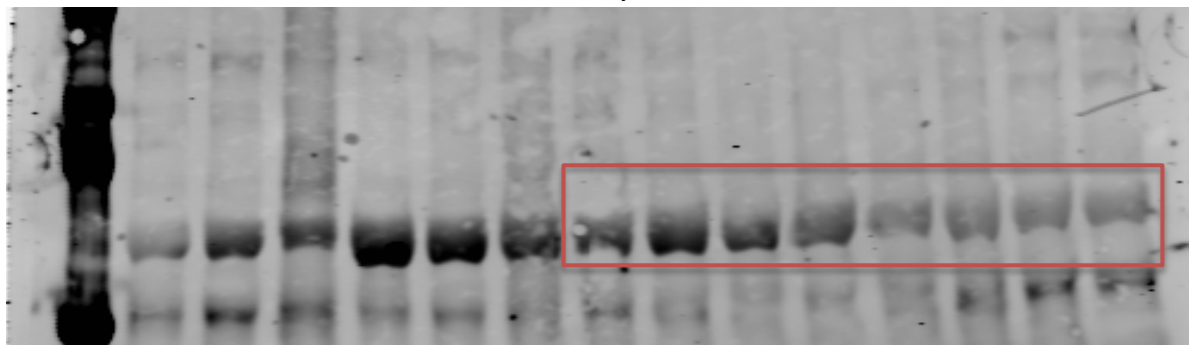

Actin

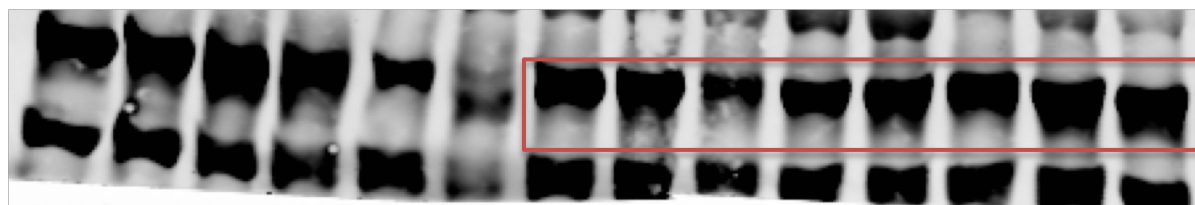

Supplement: Supplementary file 5 — Source Data for Figure 2 [file EMMM-11-e9976-s003.pdf]

**Figure 5B**

Notch

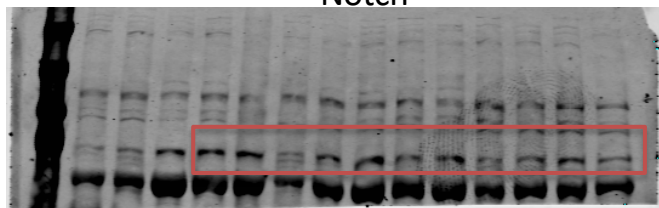

pEGFR

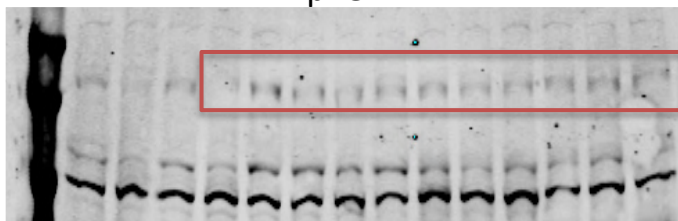

Pro/mature Nrg1

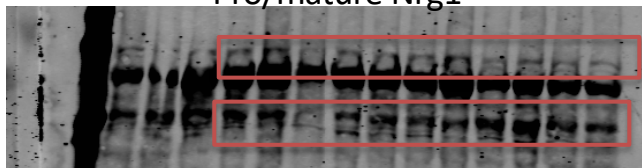

EGFR

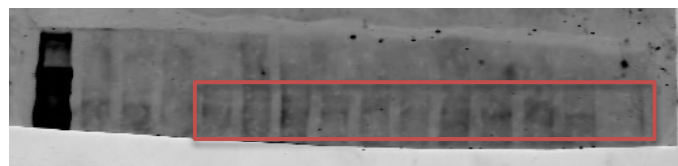

Pro/mature TGF

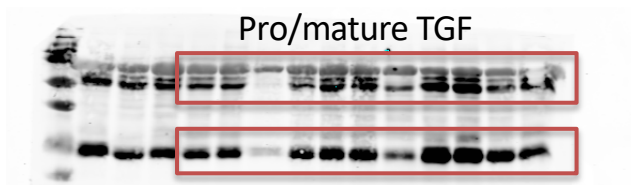

Actin

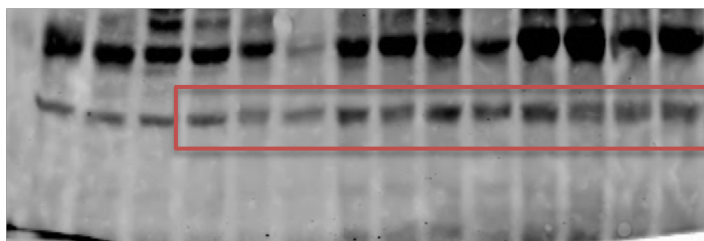

pErbB3

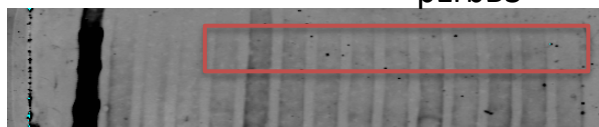

ErbB3

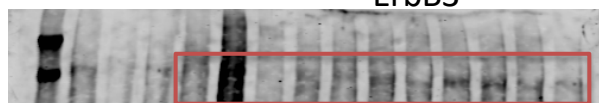

Figure 5F

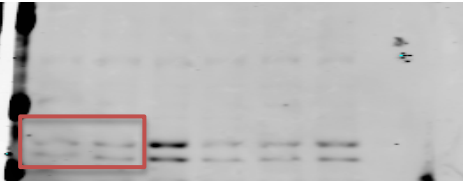

pERK1/2

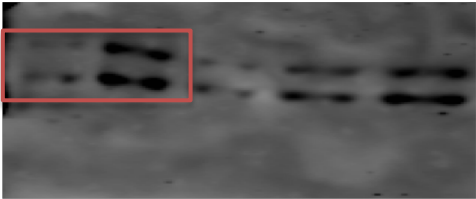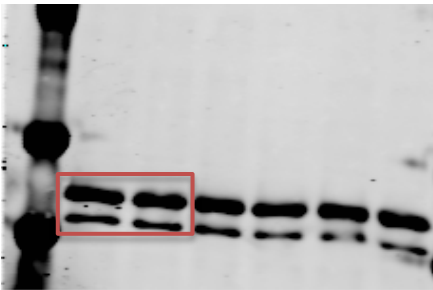

ERK1/2

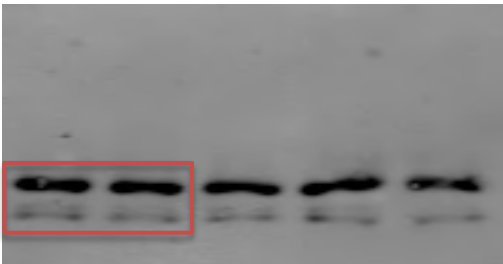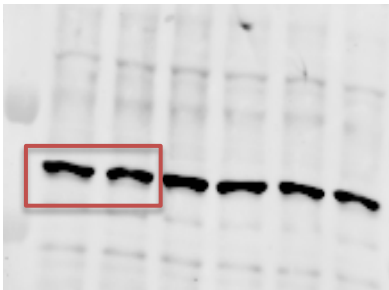

Actin

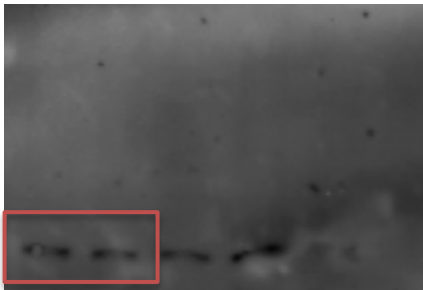

Supplement: Supplementary file 7 — Source Data for Figure 5 [file EMMM-11-e9976-s005.pdf]

Figure 6F

pADAM17

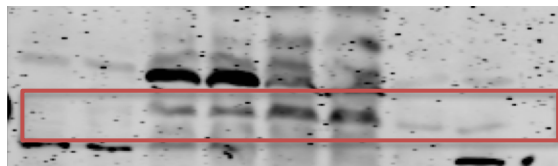

pp38

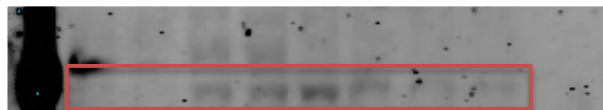

Pro/mature ADAM17

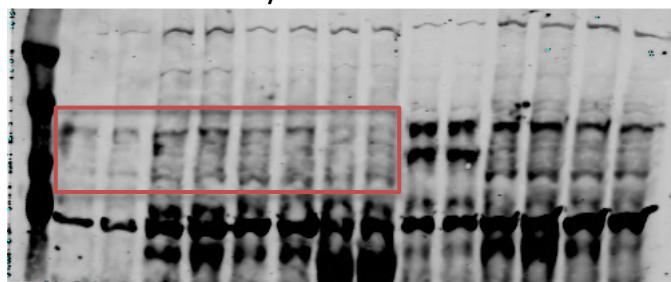

p38

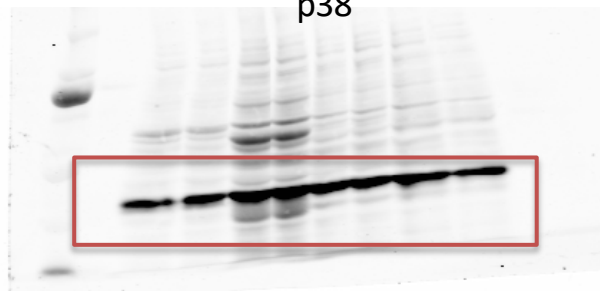

pERK1/2

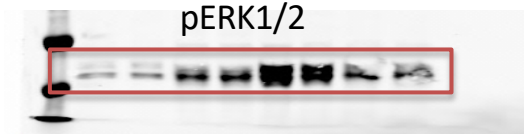

Actin

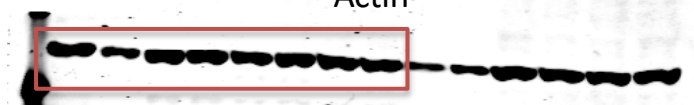

ERK1/2

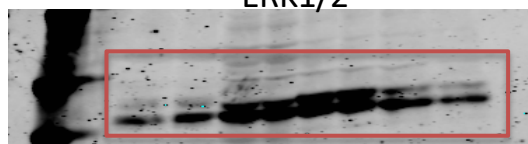

Figure 6Q

Pro/mature NRG1

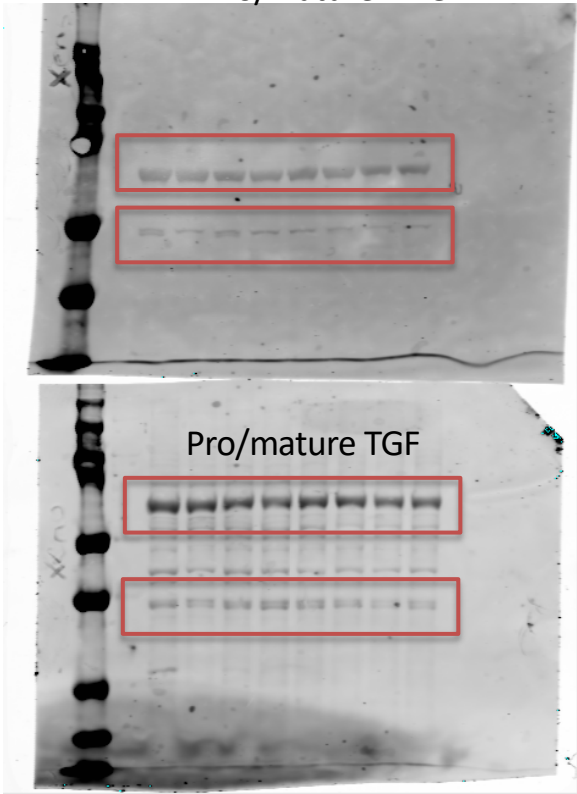

Actin

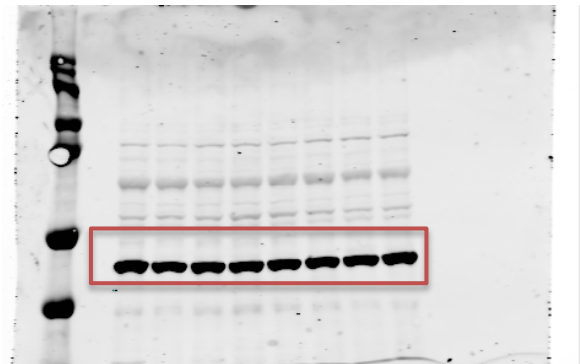

Supplement: Supplementary file 8 — Source Data for Figure 6 [file EMMM-11-e9976-s006.pdf]

Figure 7N

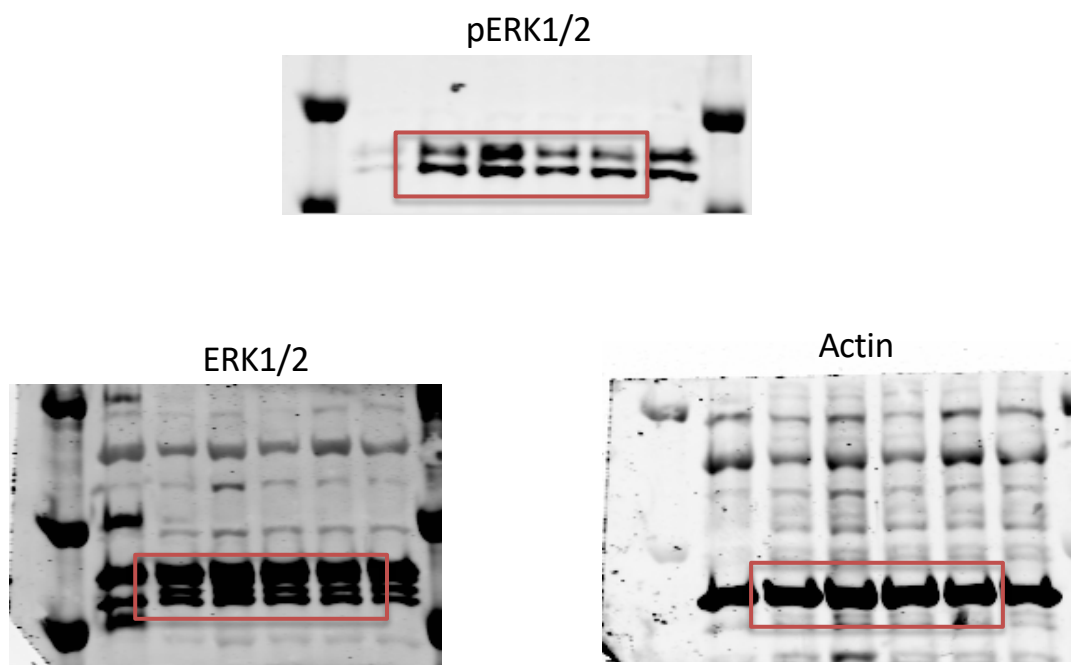

Supplement: Supplementary file 9 — Source Data for Figure 7 [file EMMM-11-e9976-s007.pdf]
