# Supplementary material for: ADAM17 selectively activates the IL‐6 trans‐signaling/ERK MAPK axis in KRAS‐addicted lung cancer
Source: EMBO Mol Med. 2019 Mar 4;11(4):e9976. doi: 10.15252/emmm.201809976 (PMC6460353; doi:10.15252/emmm.201809976)
Supplement: Supplementary file 6 — Source Data for Figure 4 [file EMMM-11-e9976-s004.pdf]

Figure 4C

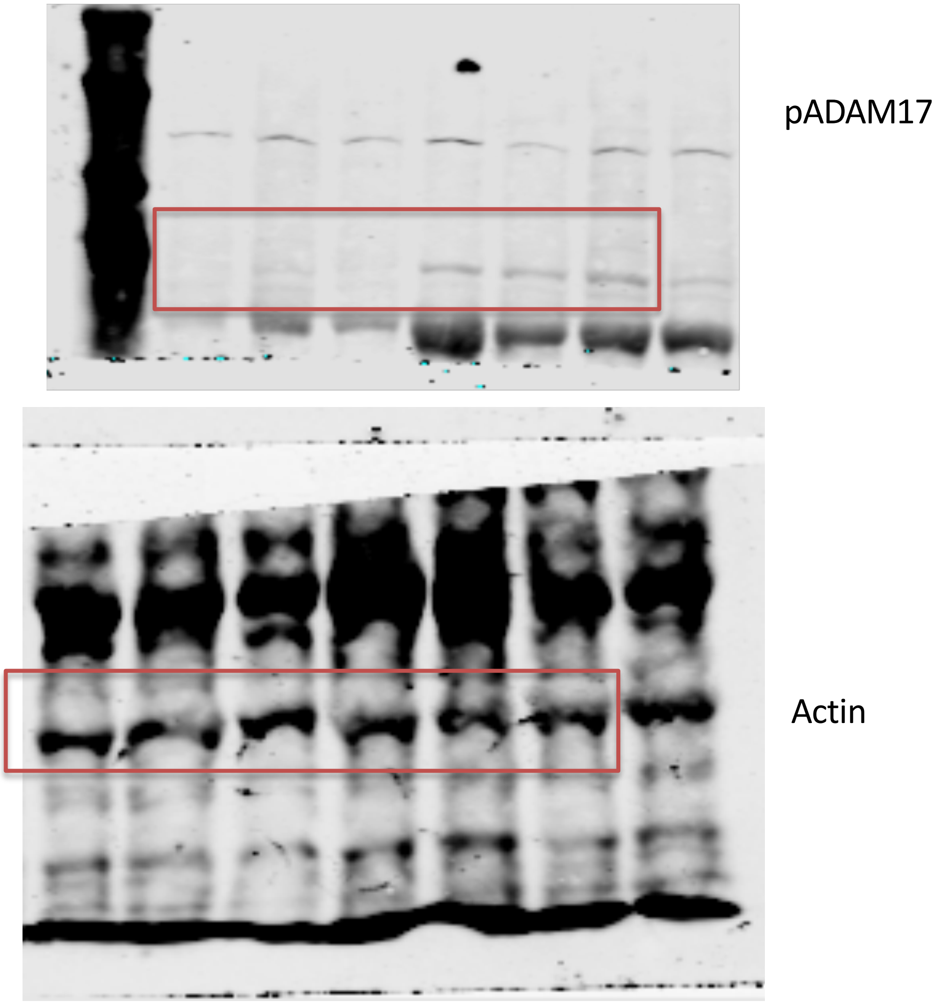

Figure 4E

pp38 MAPK

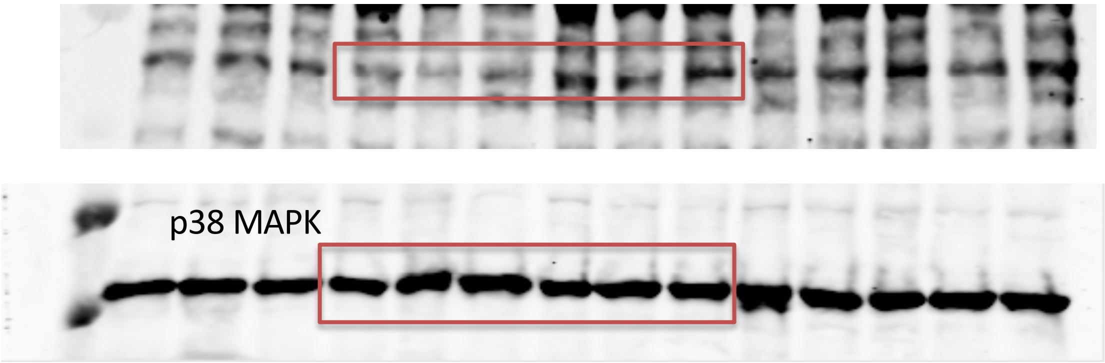

The lanes 4-9 from the left in the above blots are reproduced and used in Fig 4L.

pERK1/2

ERK1/2

Actin

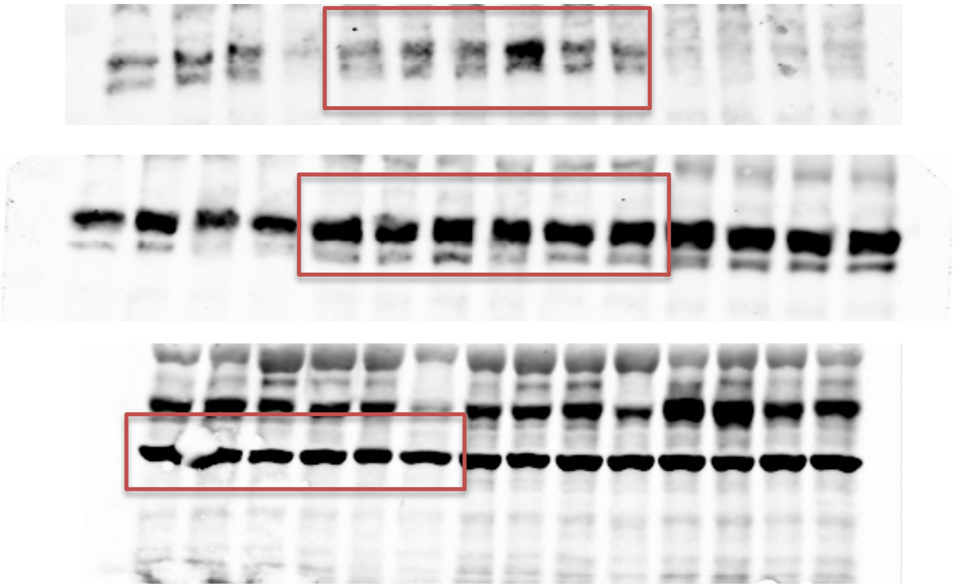

Figure 4I

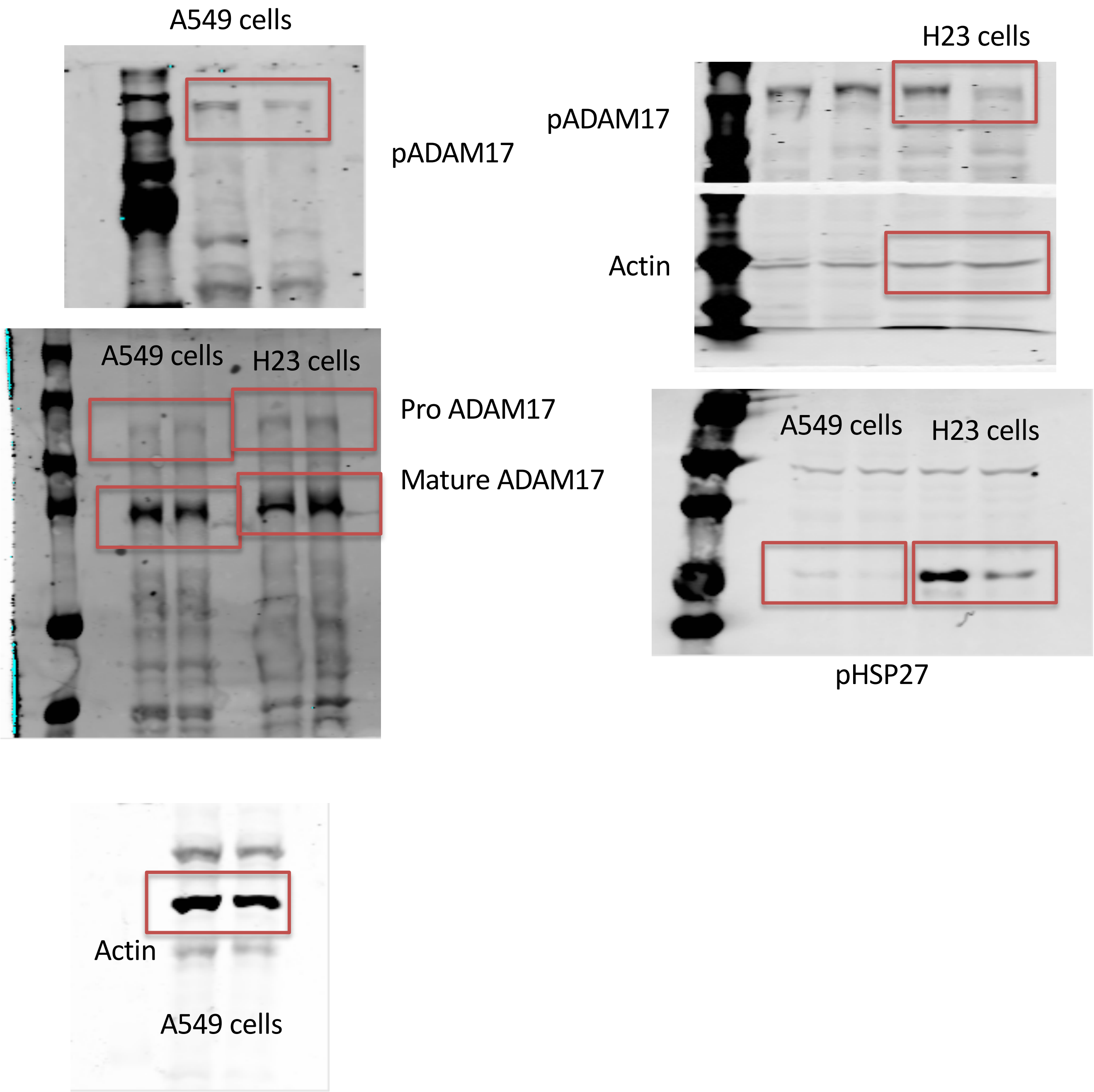
A549 cells
pADAM17
H23 cells
pADAM17
Actin
A549 cells
H23 cells
Pro ADAM17
Mature ADAM17
A549 cells
H23 cells
pHSP27
Actin
A549 cells

Figure 4L

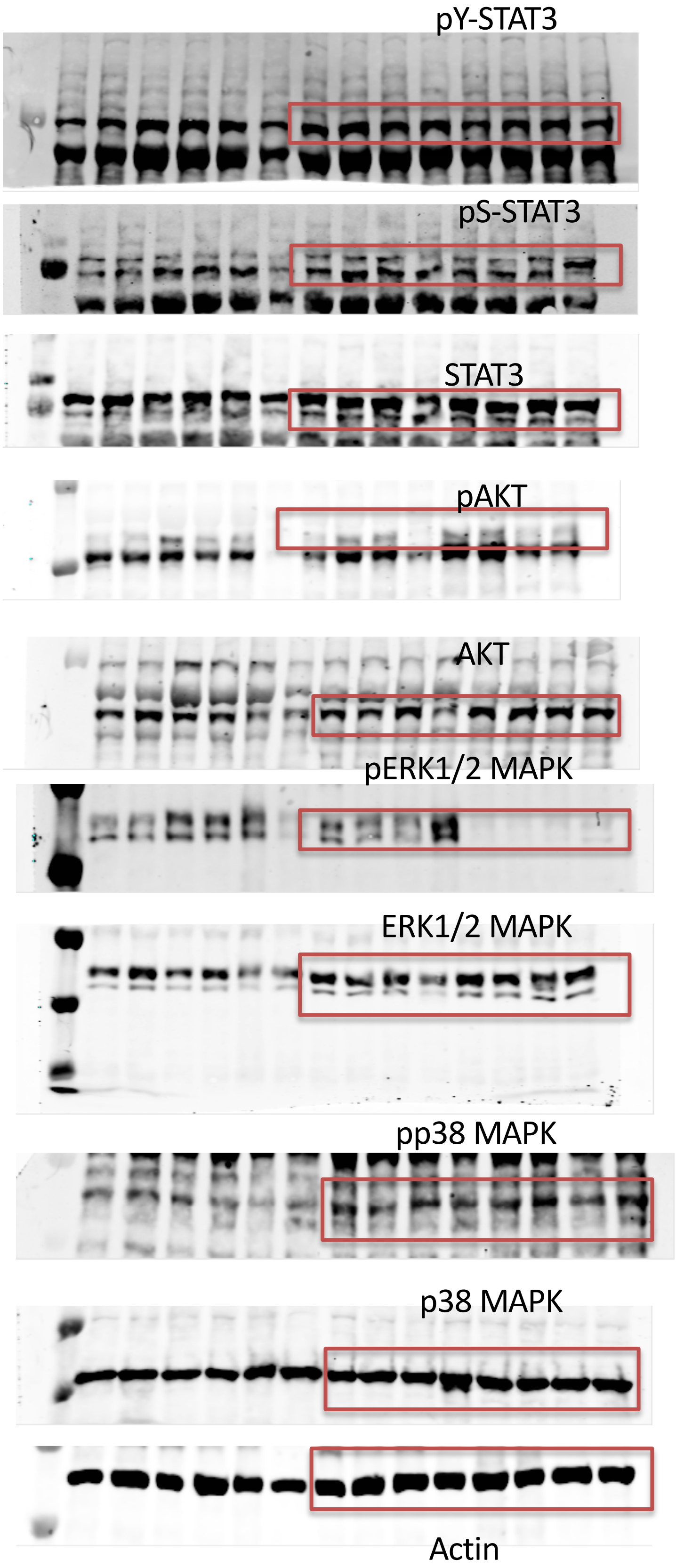

The lanes 4-9 from the left in the actin blot are reproduced and used in Appendix Fig. 2S (B)
